# “A man’s gonna do what a man wants to do”: African American and Hispanic women’s perceptions about heterosexual relationships: a qualitative study

**DOI:** 10.1186/1472-6874-13-27

**Published:** 2013-05-24

**Authors:** Eleanor McLellan-Lemal, Lauren Toledo, Christine O’Daniels, Olga Villar-Loubet, Cathy Simpson, Ada A Adimora, Gary Marks

**Affiliations:** 1 Division of HIV/AIDS Prevention in the National Center for HIV/AIDS, Viral Hepatitis, STD, and TB Prevention, Centers for Disease Control and Prevention, Georgia, USA; 2 ICF International, Atlanta, Georgia, USA; 3 Carter Consulting, Inc., Atlanta, Georgia, USA; 4 University of Miami Miller School of Medicine, Miami, Florida, USA; 5 University of Alabama at Birmingham, Birmingham, Alabama, USA; 6 University of North Carolina at Chapel Hill, Chapel Hill, North Carolina, USA

**Keywords:** Heterosexual women, Relationship schemas and scripts, HIV risk perceptions

## Abstract

**Background:**

HIV prevention efforts have given limited attention to the relational schemas and scripts of adult heterosexual women. These broader schemas and scripts of romantic and other sexual liaisons, partner selection, relationship dynamics, and power negotiations may help to better understand facilitators and barriers to HIV risk-reduction practices.

**Methods:**

We conducted exploratory qualitative interviews with 60 HIV-uninfected heterosexual African-American women from rural counties in North Carolina and Alabama, and Hispanic women from an urban county in southern Florida. Data were collected for relationship expectations; relationship experiences, and relationship power and decision-making. Interview transcripts underwent computer-assisted thematic analysis.

**Results:**

Participants had a median age of 34 years (range 18–59), 34% were married or living as married, 39% earned an annual income of $12,000 or less, 12% held less than a high school education, and 54% were employed. Among the Hispanic women, 95% were foreign born. We identified two overarching relationship themes: contradictions between relationship expectations and desires and life circumstances that negated such ideals, and relationship challenges. Within the contradictions theme, we discovered six subthemes: a good man is hard to find; sex can be currency used to secure desired outcomes; compromises and allowances for cheating, irresponsible, and disrespectful behavior; redefining dating; sex just happens; needing relationship validation. The challenges theme centered on two subthemes: uncertainties and miscommunication, and relationship power negotiation. Gender differences in relationship intentions and desires as well as communication styles, the importance of emotional and financial support, and the potential for relationships to provide disappointment were present in all subthemes. In examining HIV risk perceptions, participants largely held that risk for HIV-infection and the need to take precautions were problems of women who differed from them (i.e., abuse drugs, are promiscuous, exchange sex).

**Conclusion:**

Underlying women’s relational schemas was a belief that relationship priorities differed for men and women. Consequently, expectations and allowances for partner infidelity and negligent behaviors were incorporated into their scripts. Moreover, scripts endorsed women’s use of sex as currency in relationship formation and endurance, and did not emphasize HIV risk. Both couple- and gender-specific group-level interventions are needed to deconstruct (breakdown) and reconstruct (rewrite) relationship scripts.

## Background

In 2009, heterosexual men and women residing in the United States accounted for 27% of new HIV infections and 68% of those infected through heterosexual sex were women, with African American and Hispanic women bearing the greatest burden [1]. Eighty-five percent of African American and 82% of Hispanic women living with HIV were infected through heterosexual sex [1]. Thus, it is plausible that after almost 30 years of safer- sex messages advocating condom use, those messages have been unheard, deemed undesirable, viewed as unfeasible (e.g., pressure from partner to forgo condoms, lack of negotiating skills or power), or irrelevant (e.g., not applicable to them given low self-perceptions of being at risk for HIV infection) or interpreted differently by heterosexual African American and Hispanic women.

To date, HIV prevention efforts have given cursory attention to gender relations and social structures and norms that frame sexual roles and individual behavior. Gender-responsive programming has been proposed by some as a new opportunity for changing the United States’ HIV epidemic [2,3]. Researchers have argued that the dominant behavioral risk-reduction models in HIV prevention do not take into account contextual and social factors that influence women’s sexual behavior [4-8]. In addition to pointing out that HIV prevention messages have ignored the influence of cultural norms and the context of sexual negotiations for women [4,9,10], research has suggested that HIV prevention information alone is not sufficient for changing sexual risk behaviors [11,12].

The literature provides ample evidence of the challenges associated with heterosexual safer-sex communication and practices for women. Shifting people’s views on condoms from being predominately a birth control method to a means of preventing HIV infection has been difficult [13], perhaps due in part to the perception among heterosexual men and women in Western/developed countries that HIV is not a heterosexual issue [14]. The literature has further proposed that unprotected sex is symbolic of intimacy, romance, and trust on the women’s part [15-17]. Regardless of potential risk in a sexual relationship, women may be disinclined to use condoms if they desire a steady relationship and they equate condoms with casual sexual relationships [18-20].

Sexual scripts provide the instructions for how to interpret and respond to sexual cues as well as how to behave sexually [21]. Within the context of personal relationships, in particular those involving regular or main partners, discussion and practice of safer sex and condom use are seen as unnecessary or in violation of the expectations and assumptions embedded within the script of heterosexual relationships [22,23]. While the sexual scripts of adolescent women, college-attending women, and women who had experienced coercive or non-consensual sex are well documented [24-36], the focus of most scripting research on heterosexual women has been related specifically to contraception negotiation, clinic attendance, condom use, exchange sex, and forced or unwanted sex [16,37-40]. Little attention has been given as to how broader relationship beliefs (i.e. relational schemas) that frame dating, romantic, sexual, and other kinds of heterosexual scripts may influence HIV risk-taking behaviors. By examining the broader social construction and re-enactment frame for heterosexual relationships, we may be able to better understand underlying facilitators and barriers to adopting HIV risk-reduction practices and consequentially identify and address sexual health strengths and flaws rooted in relational schemas and scripts of women. Presumably, expectations for how relationships are initiated, played out, and terminated are based on prior relationship experience as well as relationship insights shared by others, and relationship depictions in literature, films, and the popular media [41]. Despite the growing literature on the influence that the internet, music videos, television programming and commercials have on adolescent sexual behavior [42-44], it remains unknown whether these sources both reflect and reinforce adult heterosexual women’s heterosexual scripts or whether women use this information to model their relationships and sexual behavior.

A model of relational schemas assumes that people organize representations of past behavior and experience, which they then use to interpret and act on new experiences in their social environment [41,45-47]. In cognitive anthropology, these schemas are referred to as cultural models. Within the relational schemas or cultural models, people collectively develop, share, enact, and internalize a variety of scripts that contain a representational knowledge regarding a predetermined sequence of events and actions [45-47]. Part of this representational knowledge is descriptive and part is procedural. With descriptive knowledge, the characteristics of events, interactions, and persons can be taken apart and analyzed (deconstructed) into episodic and semantic memories of specific past occurrences, and used to make sense of and act accordingly on similar experiences [45]. Conversely, procedural knowledge refers to the repertoire of rules and skills that automatically triggers information on how to proceed to either arrive at a particular goal or move towards a desired end state. Procedural knowledge provides the if-then contingencies that guide one’s social interaction and behavior [45]. All scripts contain specifications for the role of self and all others in the social environment. When encountering a situation, people tend to resort to a particular sequence of events from a well-known situation (personally experienced or culturally emphasized) to guide their actions and decisions [48].

In this paper, we explore heterosexual African American and Hispanic women’s beliefs about relationships with the aim of describing underlying schemas for romantic and other sexual relationships as well as identifying scripts that influence their views toward and behavior in such relationships. The demographic and behavioral characteristics of women in the qualitative subsample are presented; however, emphasis is on the qualitative findings.

## Methods

Between October 2008 and July 2009, we conducted a cross-sectional epidemiological study assessing the determinants of HIV risk factors in two contiguous rural counties in north eastern Alabama, in two contiguous rural counties in eastern North Carolina, and in one urban county in southern Florida. We recruited African American women in Alabama and North Carolina and Hispanic women in Florida. Women were eligible for the epidemiological study if they: were born a female, self-identified as African American (AL and NC) or Hispanic (FL), were between 18–59 years of age (19–59 in AL due to age of majority laws in that state), reported vaginal or anal intercourse with a man in the past 12 months, were not previously diagnosed as HIV infected, were willing to be tested for HIV using rapid oral testing, were willing and able to give informed consent, and understood English (AL and NC) or either English or Spanish (FL). Findings from the epidemiological study have been published elsewhere [49].

Exploratory qualitative interviews were undertaken with a sub-set of study participants. We established a priori that 20 interviews per site were sufficient for reaching data saturation [50-53]. To ensure that we could arrive at a sample that approximated the diversity of the participants in the main study, every fifth woman taking part in the survey data collection was invited to participate in a face-to-face, audio-recorded, individual one-hour interview. Hence, the only eligibility criterion for participation in a qualitative interview was completion of the epidemiological study.

### Data collection

The study was approved by the United States Centers for Disease Control and Prevention and the United States Office of Management and Budget, as well as local Institutional Review Boards at the University of Miami Miller School of Medicine, Miami, Florida and the University of North Carolina at Chapel Hill, Chapel Hill, North Carolina. Our Alabama collaborators, a local community-based organization, relied on the CDC IRB for review and oversight of its human subjects research associated with this study. Women provided written informed consent to take part in the interview and to have the interview audio recorded before data collection was initiated. Participants in Florida were given the option of informed consent either in English or Spanish.

Qualitative data collection was initiated approximately one month into the epidemiological survey data collection at each site (recruitment and data collection process is described in greater detail in [49]). Four recruitment approaches were used: venue-based, advertisements in locally posted flyers, a participant-referral incentive system similar to respondent-driven sampling [54], and word-of-mouth referral without incentives. Recruitment venues included beauty salons, laundromats, shopping centers, churches, local community organizations, educational/training facilities, bars/clubs, transportation centers, and health clinics.

To reduce the potential for participant burden, all qualitative interviews were scheduled to occur no earlier than 24-hours and no later than 30 days following survey data collection. At each site, the one-on-one interviews were conducted in a private room at a field office. Field staff trained in qualitative interviewing were matched with participants on race/ethnicity and gender, and study staff at the Florida site were bilingual (English and Spanish). Participants in Florida were given the option of completing the interview in either English or Spanish. All participants received a $30 gift certificate for completing the qualitative interview.

Demographic and selected risk data collected in the epidemiological survey were used to characterize our qualitative sample. Demographic data included age, education, marital status, income, and employment status. Behavioral variables included substance use, HIV testing, sex exchange, STI history, pregnancy, number of male sexual partners, unprotected vaginal and anal sex in the past 12 months, and history of violence and unwanted or forced sex. Women also provided information on whether she “knew or suspected” that her partner had other sexual partners.

Interviewers received training on the intention of all interview guide items along with a detailed intention version of the interview guide to help ensure that appropriate probe questions were asked. However, because semi-structured interview guides allow participants to talk about what they see as relevant and important, flexibility during interviews permitted information emphasized by a participant to be adequately covered. A copy of the semi-structured interview guide is provided in Additional File 1.

### Data analysis

Descriptive statistics were used to characterize our study sample demographically and behaviorally. Audio recordings were transcribed following a standardized transcription protocol, and transcripts were verified for accuracy against audio recordings [55]. Interviews conducted in Spanish underwent a simultaneous English translation and transcription process; interviews conducted in English were transcribed verbatim. A computer-assisted textual data analysis was undertaken. Iterative codebook development and analysis [56], which involved a constant comparative approach, guided the coding process using AnSWR: Analysis Software for Word-based Records [57].

The qualitative data analysis presented here focused on three major domains of inquiry: (1) relationship expectations, including preferred partner characteristics and types of relationships; (2) relationship experiences, including dynamics, cross-gender communication, and relationship power and decision-making; and (3) views on who is and is not at risk for HIV, and why. Information for each of three domains of inquiry of interest was systematically drawn from multiple interview guide items in the post-coding examination of themes and patterns and resulted in a reorganization of data so the analysis was driven by the participants’ perspectives and language (i.e., an emic focus) rather than those inadvertently created by the research team through the interview guide (i.e., an etic focus) [58].

We applied a social construction framework using sexual scripting theory to analyze and interpret the interview data. Social learning and enactment of sexual scripts from Simon and Gagnon’s [59] sociological theory approaches sexual scripting from three distinct levels: cultural scenarios provide role instructions/guidance (entry, performance, and exit); interpersonal scripts take a person from just being trained on his or her role to being able to ‘re-write’ cultural scenarios using relevant materials to create context-specific scripts; and intraphysic scripts enable an individual to abstractly connect personal wishes and desires to social meanings (i.e. construct a unique personal script).

Textual data were segmented (broken up into meaningful units) and assigned structural codes that identified both the domain of inquiry and the specific interview guide question corresponding with the text [60]. Text associated with each interview guide item was assigned a structural code by the second author and verified by the first author. Structurally coded text was then split up into coding “batches” for content-based coding. For each batch, the first two authors independently performed content coding and assessed intercoder agreement using Kappa scores. The intercoder assessment was part of the analysts’ debriefing process as opposed to an objective rater assessment [61]. The assessment was primarily carried out to ensure that all relevant data were coded and that all codes were applied consistently within and across transcripts. An overall Kappa score was generated for each coding batch using the participant as the unit of analysis. In addition, code-specific Kappa scores were produced at the text segment level. All coding batches resulted in an overall Kappa score ≥80. After each batch assessment, coders jointly reviewed and resolved differences and made codebook modifications where necessary. Previously coded text was reviewed to ensure that coding consistency was maintained and that application of new codes or modified codes (i.e., concepts merged or split up) still fit the text. At the end of the coding process, all discrepancies were resolved and a final AnSWR coded dataset produced.

Consideration was given to undertaking separate analyses for African American and Hispanic women. However, early in familiarizing ourselves with the transcripts, developing the analysis codebook, and in performing preliminary analysis, it became apparent that the most notable analytical difference was stylistic rather than content-related. While a systematic linguistic analysis goes beyond the aims of our study, we, nevertheless observed that African American women took an individualistic, direct communication approach. Hispanic women emphasized the collective and communicated more eloquently (and philosophically) about gender roles and relationship dynamics.

## Results

### Sample characteristics

All women who were invited to take part in the qualitative interview agreed to participate. As shown in Table 1, the median age of the qualitative interview participants was 34 years (range 18–59), 34% were married or living as married, 12% held less than a high school education, 39% had an annual household income of $12,000 or less, and 54% were employed. Ninety-five percent (19/20) of the Hispanic women from FL were born outside the U.S. (data not shown). The median age when they first moved to the U.S. was 25 (range 11–55 years). Of those born outside of the U.S., 42% were from a South American country, 36% from Central America, 15% from Cuba, and 5% from the Dominican Republic.

**Table 1:** Demographic and behavioral characteristics of qualitative participants, five counties in the southeastern US, 2008-09

	**Participants completing qualitative interview **^**a **^**(n = 60) n/N (%)**
**Demographic:**	
Race/ethnicity	
African American (AL & NC)	40/60 (67)
Hispanic (FL)	20/60 (33)
Median age (range in years)	34 (18–59)
Age categories (years)	
18-24	14/59 (24)
25-34	16/59 (27)
35-44	15/59 (25)
45 and older	14/59 (24)
Education	
Less than high school graduate	7/59 (12)
High school graduate/GED	20/59 (34)
Tech/trade school graduate; some college	20/59 (34)
College graduate	12/59 (20)
Marital status	
Single, never married	18/59 (31)
Married or living as married	20/59 (34)
Separated, divorced or widowed	21/59 (36)
Annual household income	
Less than $6,000	8/55 (15)
$6,001-$12,000	13/55 (24)
$12,001-$24,000	20/55 (36)
More than $24,000	14/55 (25)
Employed full or part time	32/59 (54)
**Behavioral:**	
Binge drinking past 30 days^b^	11/59 (19)
Non-injection drug use past 12 mos	
None	47/59 (80)
1 drug	7/59 (12)
2 or more drugs	5/59 (8)
HIV testing past 12 mos	15/59 (25)
Diagnosed with a sexually transmitted infection (not HIV) past 12 mos	8/59 (14)
Ever pregnant	49/59 (83)
Number male partners past 12 mos	
1 partner	35/59 (59)
2 partners	15/59 (25)
3 or more partners	9/59 (15)
Unprotected vaginal intercourse past 12 mos	56/59 (95)
Unprotected anal intercourse past 12 mos	15/59 (25)
Reported partner(s) had concurrent female partner(s) in past 12 mos	25/58 (43)
Ever physically hurt or threatened by male sex partner	14/58 (24)
Ever experienced unwanted or forced sex	20/58 (34)
Ever engaged in exchange sex	7/58 (12)

*Note:* Sample sizes fluctuate slightly for some variables due to missing data. Some percents do not sum to 100 due to rounding.

^a^ Complete survey data not electronically captured for one qualitative interview participant.

^b^ 5 or more alcoholic drinks in single day.

Nineteen percent of the qualitative sample reported binge drinking (5 or more drinks in a single day) in the past 30 days and 20% had used one or more non-injected illicit drug(s) in the past 12 months (AL: 37%; NC: 20%; FL: 5%).

Approximately 72% reported that they previously had been tested for HIV infection and, of these, 25% had been tested in the past 12 months. Excluding HIV, 14% indicated that they had a sexually transmitted infection (STI) in the past 12 months (AL: 21%; NC: 20%; FL: 0%). In the preceding 12 months, 40% reported having had more than one male sexual partner (AL: 63%; NC: 40%; FL: 20%), 95% engaged in unprotected vaginal sex (AL: 95%; NC: 100%; FL: 90%) and 25% engaged in unprotected anal sex (AL: 26%; NC: 15%; FL: 35%). Forty-three percent indicated that one or more of their male partners in the past 12 months were “probably” or “definitely” having sex concurrently with other women. Overall, in their lifetimes 24% had been physically hurt or threatened by a male sex partner at least once (AL: 21%; NC: 25%; FL: 25%), and 34% of the women reported that they experienced unwanted or forced sex at least once (AL: 37%; NC: 35%; FL: 30%). Overall, 12% (AL: 21%; NC: 10%; FL: 5%) indicated that they had used sex to get things that they needed or wanted in the past 12 months.

Our qualitative analysis identified two overarching themes related to African American and Hispanic women’s relationship beliefs and experiences. The first highlighted the contradictions between women’s expectations and desires for their relationships and life circumstances that sometimes negated such ideals. Within this theme, we discovered six subthemes: a good man is hard to find; sex can be currency used to secure desired outcomes; compromises and allowances for cheating, irresponsible, and disrespectful behavior; redefining what dating means; sex just happens; needing relationship validation. The second theme focused on relationship challenges and had two subthemes: uncertainties and miscommunication, and negotiating relationship power. Gender differences in relationship intentions and desires as well as communication styles, the importance of emotional and financial support, and the potential for relationships to provide disappointment were recurrent within all eight subthemes. Lastly, in examining HIV risk perceptions we found risk for HIV infection was largely viewed as a problem faced by others, who were seen as being “not like” the participant.

### Ideal vs. Real: the relationship quandary

#### A good man is hard to find

Participants indicated that the ideal man respected, loved, and accepted them and their children, was faithful, and could be counted on to provide emotional (“was there for her”, “backed her up”, “encouraged her”) as well as financial support. Some participants emphasized that it was important to find a man that they got along with well, could talk to about anything, who shared similar interests or goals, and who spent time with them. Others focused on having a man who had his life together (“knew where he was going with his life”), or was stable and responsible. Such men were portrayed as honest, trustworthy, educated, employed, having high self-esteem, working toward achieving their goals and aspirations, and being religiously or spiritually observant. Participants, in particular those from AL and NC, made it clear, however, that partner availability often outweighed desired partner preferences. It was viewed that “a good man was hard to find” given difficulties that men had with fidelity or commitment as well as shortages of available men. One participant stated:

“Now every time my friends talk about something that happen they always talk about the man done cheated but they'll stay with him cause she'll be like I can't find no another good man and I just be like that ain't no good man (laughs).”

Another participant further explained:

“Some woman, I feel, you know, because they have a man, he may be a good man to them in their eye sight as far as a provider but he still have this little thing on the side, you know, one woman ain't enough for him. I've seen women put up with them being cheaters, a husband or a friend or their partner cheatin' on them, just to keep what they feel like is a stable life, you know, because he's a provider….But just cause you got somebody to put britches on don't necessarily make him a man.”

Participants held that men in turn sought an independent, good-looking female partner who was either only interested in a sexual relationship with no emotional commitment or offered willingness to commit emotionally and sexually only to him as well as manage the household and do the childrearing. As explained by one participant,

“Well, her role is, I would say if they have children to make sure she's a good mother first. Her role is to take care of the domestic things. I know that's kinda cliché is the word I guess but I think her role is to make sure she does the best to make sure that her man is happy so she won’t have to worry about another woman doin’ that. Which [that doesn’t] always work either because a man’s gonna do what he wanna do....Make sure that, you know, your man has, make sure food is in the house, make sure that the clothes are clean and like I said all the domestic things.”

#### Sex as social currency

Sex was described as having social, emotional, physical, and economic value (i.e., currency). Participants indicated that sex could be withheld or offered to achieve desired relationship outcomes. In situations where a committed relationship was not desired or attainable by a woman, participants indicated that women sought sexual partners who could provide money and other material benefits as well as sexual satisfaction. As one NC woman commented,

“I look for honesty. He has to [be] very confident of himself….[Other women] should be looking for the same attributes, but they just look for someone who can take care of them financially….A lot of them are looking for money. I can’t think of the word I’m looking for, but they basically lookin’ for somebody to take care of them. They don’t really think about what it is that they’re bringin’ to the table and what do I have to offer this man. A lot of them are all about what can this man do for me.”

#### Compromises and allowances

Participants indicated that heterosexual sexual relationships are stable when couples are compatible, spend time together and demonstrate acceptance, love, and respect for one another. Equal decision-making, commitment, and communication were viewed as being central for a relationship to endure. Conversely, deception (i.e., not being upfront), infidelity, and partner violence created conflict and instability. Disrespect and poor communication were viewed as early indicators (“red flags”) of and subsequent contributors toward relationship problems. Participants held low, if any, expectations that a sexual partner would remain sexually faithful and acknowledged that having multiple sexual partners was not unique to men; however, references to women cheating were not common. Participants repeatedly talked about how other women were likely to put up with a cheating man. They explained that such women preferred “being blind” to infidelity; thus, not knowing or not being able to prove that a man was cheating, required no action on the woman’s part and less likelihood for relationship conflict.

Mention of men’s inability or unwillingness to practice sexual fidelity was presented in conjunction with other role performance failures (e.g., inability to meet financial obligations, unequal division of child rearing responsibilities or other household tasks). Participants indicated that there was an expectation that a man will take the initiative to perform his role and handle his responsibilities but *“if there’s something needs to be done, [a woman] will get out and just do it.”*

Participants reported that women also sometimes tolerated verbal abuse and physical violence. A number of participants spoke of their personal experiences with intimate partner violence. One participant explained that:

“Some women they get beat and they still stay. Some women get beat and they leave. And I got beat and I left.”

Another indicated that:

“You gotta kind of put up with it for a little while. Because I've been there…. If a relationship is not safe to get out, she still might [take] a while to get out because sometime[s] you have to. That's why it comes in, that compromising. You don't want to compromise on no bad thing like that. But if you know leaving is gonna make it even worse [for] you, you’re gonna see a better way out.”

Overall, participants talked at length about the importance of trying to work through problems. Some participants commented that they personally tried to work through a partner’s infidelity as long as he strove to be financially responsible (able to provide for her and her children). All viewed men as having low tolerance for women who cheated or were unable to carry out their expected traditional mothering, nurturing, and domestic caretaking roles. Participants commented that in such situations, men are more likely to leave or find another woman than to try to work things out.

#### Redefining what it means to date

Idealistic expectations (“how it should be”) about relationship development were commonly expressed by participants. Descriptions of personal relationship experiences that failed to meet those expectations were offered by some of the participants. References to dating were largely in the context of relationship histories in their younger days. Participants indicated that in the present day, conventional dating was less common; a man might call or drop by, but that he did not take a woman out to dinner or a movie. This interaction was described typically as involving alcohol use and sexual activity. One participant explained:

“Well, people don’t date like they used to. They used to date a long time [ago]. Now… it’s not even considered a bad thing if you meet somebody and two, three days later you have sex with them. It used to be, you know, you didn’t do that. You was considered, you know, nasty or whatever, but now it’s the thing. You meet somebody, it’s like an instant attraction, so you like them, you don’t even think about this might not work. You try to put everything aside that you already see that is wrong, red flags jumping up, and you just hit the bed. There is no dating and dinner, and flowers, and candy, and it’s nothing like that, no planning whatsoever, no finding out about their background, it’s let’s get it on.”

Participant-generated “if-then” contingencies were described for six romantic courtship scenarios (general public encounter, bar/club encounter, online encounter, introduction by a common source, friends to lovers, rational choice) whereby interaction was typically initiated by men. Across these six courtship scenarios, five common themes on courtship evolution were found: connecting, appraising intent, having sex, bonding, and outcomes (Additional File 2). *Connecting* included a range of behaviors. Depending on the type of courtship scenario, connecting involved a passive role whereby a physical place (e.g., bar or nightclub) or social situation (e.g., friend of a friend) created an interaction opportunity versus cases in which the search for a man was more active (e.g., seek men who share common interests or have particular traits/characteristics). A*ppraising* involved strategies for both determining a man’s relationship intentions and for getting to know him. Participants described sometimes relying on second-hand information (e.g., reputation in the community, family background) to provide insights on any relationship patterns that could be helpful in figuring out if a man was just looking for sex. For the most part, participants described (a) “game-playing” strategies that included delaying subsequent contact with a potential partner (e.g., waiting to initiate or not returning phone calls) to see if they maintained interest; (b) gauging the degree of physical chemistry present; and (c) instinctual feelings that they “were meant for one another” (e.g., love or passion at first sight). The *having sex* theme focused on the timing of the initiation of sexual interaction. In some instances this happened shortly after meeting or once an interest in developing a relationship was made known by one or both parties. In scenarios where sex occurred shortly after meeting, women often described a physical connection or attraction that fueled the encounter, despite the inability to ensure the relationship would continue. Other women described delaying sex (ranging from hours to weeks) until they could develop a bond or gauge the level of commitment their partner was willing to provide. *Bonding* referred to whether or not an emotional commitment resulted. With the exception of starting out as friends or being introduced by a common source, most courtship scenario *outcomes* were depicted as not resulting in a committed relationship and ending in disappointment.

Boundaries between romantic and non-romantic sexual relationships were perceived to be clear and well-defined. Romantic relationships were described as involving a range of sexual and non-sexual overtures that validated a woman’s belief that a man was emotionally prepared to commit to her. In the idealized committed relationship, men and women took time to get to know one another, spent time together and met one another’s families regardless of when sex was introduced as part of the relationship. If sex occurred immediately or shortly after meeting, concepts of love at first sight, being drawn to him, and having a strong chemistry helped explain the behavior. In contrast, non-romantic relationships were primarily about having sex where emotional ties were neither expected nor fostered by one or both parties. In talking about non-romantic sexual relationships, participants spoke of sexual encounters consistent with what has been presented in the literature as well as the popular media as the *hook-up,* the *booty call, and friends with benefits* without necessarily using these terms. One participant shared the following perspective:

“Well, they have friend relationships and you don’t find too many of those when me and a guy could just be friends and we’re just friends with no benefits. Now these days they have different words as cuddy buddy, the friend that you have sex with or you know, a bus’ it baby, you know, friends that they have sex with. So it’s basically with guys and females it’s two relationships, you’re either their friend or their girlfriend. Well three, their friend, their girlfriend, or their side girl [cuddy buddy].”

Although women could and did maintain non-romantic relationships with men that did not involve sex, such relationships were considered rare (as also shown in the quote above). Participants largely viewed completely platonic friendships between men and women as improbable. FL participants were more likely to talk about platonic and familial relationships between men and women as indicated by the following:

“Well, I think that the majority like to share in the sexual relationship….but there are some friendships that also, there are some men and women who can have the type of relationship where it’s a friendship, of sharing like going to the movies, go to the discothèque with their friends and it can simply be that there isn’t an interest…. But it can be a friendship and not necessarily has to be of the objective being about sex.”

#### Sex just happens

Overall, participants acknowledged that the phases that men and women should go through before having sex had changed from that of their parents’ generation, where sex was perceived as less casual. These days, having sex was generally described as just happening. It was viewed as being spurred by physical, emotional, and psychological (e.g., low self-esteem, fear of not being accepted or loved) feelings. In addition, it was reported that sex was typically initiated by men. Participants uniformly acknowledged that while men were always ready to have sex, it was women who actually controlled when the first sexual encounter occurred as well as the frequency of on-going sexual activity. Setting the sexual pace, however, was viewed to come with a risk. A relationship might not continue after the initial sexual encounter if sex took place before a man and a woman had gotten to know one another well enough. Some mention was made of the potential risk for STIs, including HIV, if one did not know a sexual partner’s background and sexual history. Participants indicated that decisions about sex, including whether or not condoms should be used, were seldom talked about, but were instead made in the heat of the moment. A participant explained:

“I would say they just happen, I mean I don’t think that you really talk about it I just think whatever happens, happens.”

#### Validation

Within a dyadic partnership, participants indicated the importance of both parties viewing themselves as a committed couple, and presenting themselves in the community as such. This signaled to the woman as well as to others, that a couple has a strong bond (i.e., united as one, love one another, can talk about anything, make each other happy). Incidentally, AL and NC participants observed that marriage and long-term relationships were not common within their social networks.

### Relationship challenges

#### Uncertainties and miscommunication

Heterosexual sexual relationships were often characterized by uncertainty (e.g., one party had greater investment in the relationship, dissolution was likely but the timing was unknown) and a high degree of tolerance for men’s negligent behavior, including, as previously mentioned, a failure to remain faithful or provide financial or emotional support. As explained by one FL participant, *“we tolerate the things we don’t like in the conquest”.* In general, women perceived men as principally interested in getting a woman to have sex with him as soon as possible (i.e., upon meeting, on the first date), and that men would even say that they were looking for a relationship although they really did not want one (i.e., they only want sex). Participants indicated that when a man and a woman are interested in developing a relationship, they are typically on their best behavior.

Cross-gender communication was identified as critical in establishing a good relationship, but viewed as being seldom achieved. This was primarily attributed to men and women holding different relationship priorities. Men were thought to be interested primarily in having their sexual and other physical needs met (i.e., having someone else take care of the cooking and cleaning), while women were seen as being invested in emotional attachment and support. The idea that women love more or that women love harder was presented. In general, men were depicted as detached, uninterested in commitment, and unable or unwilling to talk about their feelings. Women, in contrast, were described as needing to talk about their feelings as well as the mundane events and people in their daily lives, but refrained from such discussions to avoid conflict or further detachment by the man. Non-verbal communication (e.g., making eye contact, touching), flirtatious behavior, and romantic gestures (e.g., sending flowers, preparing a meal) were used to both express initial and sustained interest in the other party. Participants perceived that women who made themselves too readily sexually available (comes off too easy or too strong) risked getting a bad reputation. Some women indicated that they were comfortable talking to their partners about sex, while others indicated that having such discussions was extremely difficult.

#### Negotiating power

Overall, participants favored dyadic partnerships that strove towards egalitarianism whereby financial, household, and child-rearing responsibilities were shared 50/50, yet readily acknowledged that they were prepared to step in to take care of things that a partner was neglecting. Participants indicated that this “just do it” attitude demonstrated a women’s ability to be independent and self-sufficient; when a man failed to act, it was up to the woman to make sure that things got done.

Participants talked about situations where one party, namely the man, held control in the relationship. Some participants indicated that a woman had to stand up for herself to avoid having a man think that he could “*walk over her*”. Others stressed that the idea of being controlled by a partner due to his insecurity, jealousy, and mistrust (sexual and financial) often led to the man believing that his permission was needed for a woman to do things and that he had the right to enforce such rules. While not restricted to the FL women’s experiences, the quote below helps illustrate how conflict and violence were discussed as potential risks of going against a partner’s wishes:

“....that’s when the fighting begins....The Latina woman waits for the Latino man. The Latino man is like that, he feels secure when the Latina woman waits for him at home....Any Latina woman who has a lot of time with her partner and it’s a serious relationship, she never goes here or there unless she tells her husband and almost every time she waits for her husband to go. Whatever she has to do, she waits for him.”

### HIV risk perceptions

#### It’s somebody else’s problem

In talking about persons at risk for HIV infection, participants seldom included themselves in the risk categories they identified. Statements that a participant was “not like those women” or that others were “worse than me” were common. Youth (persons 12–18 years of age), drug users, and sex workers were commonly identified as being at highest risk. When asked specifically about African American or Hispanic women, participants reported that having multiple partners, not using condoms or having a partner who had multiple sexual partners increased their chances of getting infected. Participants indicated that African American and Hispanic women could reduce their risks for HIV-infection by using condoms, receiving HIV education, limiting one’s sexual partners, as well as setting and sticking to personal limits on what they would and would not do sexually regardless of a sexual partner’s wants or demands.

## Discussion

Shifts in traditional heterosexual sexual scripts whereby women were expected to be passive recipients of men’s sexual interest, have been observed since the early 1960s [62]; yet, residual elements of these traditional scripts may persist. Our findings show that traditional gender roles were embedded in relationship expectations and experiences for women in our sample. This may help explain women’s attitudes and behaviors in their relationship dynamics, including risk-taking sexual behaviors. There may be important implications for adoption of HIV sexual risk-reduction in light of this particular finding. The literature suggests that even in situations where less traditional sexual scripts are present and increased sexual communication occurs, women may not perceive themselves to be “effective influence agents” [63]. Consistent with the “Heterosexual Script” described in the literature [43,64], our findings demonstrate that a sexual double standard exists, power dynamics favoring male initiation and decision-making are present, and perceptions that women seek commitment, while men try to avoid it are common.

The relational schema and the specific scripts (dating, communicating, negotiating, behaving sexually) guiding heterosexual sexual interaction may have a central place in perpetuating discord (real or otherwise) between what a woman wants in a relationship and what she believes the man wants. Moreover, sexual double standards in heterosexual relational schema set up expectations that sexual activity occurs within the context of a committed relationship for women and in all types of relationships for men [65]. Women in our sample described relationships that emphasized being swept away by physical or emotional intents and desires. Moreover, the idea that sex is just supposed to happen was explicitly and implicitly presented by our study participants. Participants acknowledged that this “myth of sexual spontaneity” [66] contributed to poor cross-gender communication and added to insecurities and uncertainties about relationship status on the woman’s part. Safe-sex strategies, which are contradictory to the myth of sexual spontaneity, may then get re-written into context-specific scripts that are generally held as improbable and consequentially less likely to become incorporated into a woman’s personal relations schema.

While participants emphasized that communication is essential in a healthy relationship, their accounts make it clear that communication is often lacking or constrained. A woman’s desire to remain blind to a partner’s past or current behavior to avoid threats to (a) the relationship either early in its initiation or later in its maintenance, and (b) her overall impression of her partner as well as her own ability to identify an appropriate partner may hamper sexual communication. Moreover, if in the social construction of heterosexual sexual relationship scripts, greater emphasis is placed on a woman using non-verbal and limited verbal communication to entice and hold onto a partner, little cultural guidance is provided on handling open discussions about sex, especially if it is assumed that sex will be initiated in most cases by the man, or dealing with men’s reticence to discuss their feelings or relationship expectations. Instead, the distancing and downward comparisons (i.e., evaluating other women as being in a worse situation) used to frame some of our participants’ HIV risk perceptions may help rationalize or compartmentalize sexual behaviors, especially those that occur within a committed relationship. One study found that steady dating couples who engaged in open sexual communication before the onset of first sexual intercourse had a lower likelihood of using condoms because they did not perceive themselves to be at risk for HIV/AIDS [67].

While women’s relationship schemas acknowledged the likelihood that a man will cheat and that women may tolerate partner infidelity to a certain extent, low expectations for monogamy do not appear to provide sufficient rationale for using condoms. Participants provided a thread of negative consequences for women’s health (e.g., he contracts STI or HIV from his other sexual partner, which he then transmits to her) that recognized the benefit of condom use (e.g., can help prevent her from getting a disease); however, for most, this did not necessarily translate into actual condom use. In addition, scripts failed to explicitly take into account that when women decide to end relationships that do not meet their expectations, they may then have a series of transitory dyadic sexual relationships, which could also increase risk for HIV infection.

Several studies have suggested that among lower socioeconomic class couples, sexual decisions are male dominated [68,69]. Other research has shown that women are less likely to make unpopular requests of their partners if they anticipate conflict within the relationship or fear that the relationship will end [70]. On the surface our findings suggest a similar passivity as well as some difficulty in establishing open communication about sex. However, not all decisions were described as male dominated. Women were depicted as having a critical role in handling financial, parenting, and major household plans and decisions. In situations where men fail to meet gender role expectations, participants explained that women readily come forward to fulfill these roles. For some, a desire for egalitarian relationships was internalized as women being able to *just do it all*. Resiliency and independence rather than martyrdom resonated in these experiences. Participants presented scripts that emphasized that in some situations the presence of sex as currency in relationship formation and endurance, especially where partner availability was limited. Women were described as making calculated decisions about how and when to use this currency. As has been suggested by others [71-73], we found that resource acquisition and shifting partners were viewed as important for women who engaged in relationships without having long-term intentions.

A number of HIV interventions that address social and cultural factors, including the role of power in sexual negotiation, have shown time-limited effectiveness in reducing sexual risk behaviors for selected populations of women in the United States [74,75]. Others have stressed that male involvement in safer sex negotiation is imperative to avoid reinforcing the idea that safer sex is women’s responsibility and concern [10,70]. Moreover, there is sufficient evidence that among low-income women of color, perceptions of risk and awareness of susceptibility for acquiring HIV are low [76-79]; however, recognition that a partner’s behavior increases a woman’s susceptibility of infection is present [80]. The behavioral data for our sample suggests that engaging in unprotected vaginal and anal sex with men who may have concurrent partners may be influenced by relational schemas that make allowances for male infidelity and consequentially reduce a woman’s perceptions about her risks for HIV infection.

Our findings suggest that despite clear male role expectations, women readily assumed men’s responsibilities to ensure that things did not fail through the cracks and that nagging, threatening, or placing demands on a man (which might cause him to turn to another women) were minimized. The things that a woman took on (e.g., paying bills, upkeep of the household, rearing of the children), she did because she deemed them more important to her than to him. However, when it came to what he wanted or what she thought he wanted she acquiesced even if it was not good or healthy for her to do so (e.g. puts up with his infidelity, stays when he is violent or irresponsible). Given such, it stands to reason that condoms use has to be of greater importance to women (i.e., it is worth the trouble and even the risk of a man walking away). As long as women think or know that a man is not in favor of using condoms, then women will make allowances for men not wanting to use them. For effective uptake on condoms or other female-controlled prevention technologies to occur, emphasis needs to be on figuring out what would increase their importance for women.

Preferences for a partner who was honest, trustworthy, stable, family-oriented (i.e., desire for children, including acceptance of women’s children from another union), and willing to whole-heartedly commit to the relationship was contrasted against the potential shortage of available men and unlikelihood of men’s sexual fidelity. Men and women were depicted as holding different relationship priorities: men are typically interested in a sexual union while women are predominately interested in pursuing an emotionally committed partnership. Participants identified long-term relationships, including marriage, as increasingly rare in their communities. Monogamy was viewed as unlikely given the inability of men to remain sexually faithful. While mention was made of some women being unfaithful, insufficient information was provided regarding the extent to which women play the cheating role. Emphasis was instead placed on the sexual promiscuity of such women as seeking relationships just for sex or using sex to get money or other things from men, and the risk that they would develop a negative reputation.

Changing women’s schemas and scripts for sexual relations occurring within and outside of a committed relationship may be difficult. However, we believe that there is a difference between trying to change their scripts and expanding those scripts to incorporate context-specific information that addresses both the ideal and practical elements of heterosexual sexual interaction. Based on our findings, we recommend that future heterosexual HIV preventive strategies simultaneously address men and women’s scripts. Moreover, given the larger role that culture plays in the social construction and enactment of heterosexual women’s relationship scripts and that reinforcement of these scripts that is likely to occur within women’s social networks, we advocate gender-specific, group-level HIV interventions. Sexual scripts could be incorporated into the “recognize risk” phase of *Connect: A Couples-level Intervention for Heterosexual Couples at Risk for HIV/STIs*[81]. Emphasis would be on identifying recurrent themes across a small number of vignettes and then examining similarities with past and current relationships. As part of the “commit to change” phase, the discussion would shift toward identifying ways that relationship scripts could be re-written to avoid recurrent patterns that increase risk for HIV and other STIs.

### Limitations

While our findings provide valuable insights, generalizations are neither appropriate nor possible. The process of comparing personal accounts as well as views about the motives and practices of others potentially errs toward overemphasizing similarities across our qualitative sample. Consequentially, we risk presenting African American and Hispanic women as belonging to homogenous groups rather than emphasizing that social construction of sexual relationship scripts may be influenced by other factors (e.g., economic status, education). A large number of women in our sample were of low income, resided in rural areas, and/or were foreign-born and not representative of the African-American or Hispanic to make our reference to this ethnic group consistent throughout the paper (exception would be with participant direct quotes) women residing in the southeastern United States or other parts of the country.

We recruited Hispanic women as a single subgroup rather than ensuring that our qualitative sample included a larger proportion of women representing the two largest subgroups in our survey sample, South American and Central American. We also recognize, that because the majority of the FL women were foreign-born, traditional gender roles and norms may be more pronounced in our findings than among U.S. born Hispanic women. Similarly, African-American women in our study are from rural counties from two southeastern states. Their experiences and perspective may vary from those of African-American women residing in urban regions or other rural areas in the country. We recognize that even though we present our findings by data collection sites, the potential for a comparison by race, ethnicity, culture, and geographical residence is suggested. Given that our small sample contained a number of diverse social, cultural, and historical characteristics, such a comparison would be inappropriate. Additionally, we recognize that asking participants to provide their views on what contributes to African American or Hispanic women’s risk for HIV infection does not directly tell us about personal risk perceptions. While sexual behavioral data were collected from all women in our sample as part of the epidemiological survey, the focus of this paper was not to compare women’s expectations and acceptance of partner infidelity with reported sexual behavior. Even with such an analysis, discrepancies between what a participant says and believes may not correspond with reported behaviors. Beyond findings presented here, our data does not support further examination of the disparity between women’s with men’s behavior and men’s intolerance of women’s disappointment in them. Given the gendered communication implications, future research could benefit by examining this phenomenon.

We chose to target sexually active women as opposed to focusing only on women of reproductive age. While we compared and found no notable differences for participants by age, different behavioral patterns and perspectives about relationships may exist between pre-menopausal and post-menopausal women that are not possible to explore in our data. The literature has begun to show that post-menopausal women may be at an increased risk for HIV infection given perceptions that condom use is not necessary because pregnancy is no longer an issue for them as well as the fact that biologically lower levels of estrogen can reduce the thickness of the vaginal mucosa and production of vaginal secretions which can lead to tears and abrasions [82,83]. We did not collect data on menopausal status and even if age ≥50 was used as a crude estimate, the small number of women in the post-menopausal group could present challenges in discerning salient thematic differences compared to the presumably pre-menopausal women. Lastly, we did not collect data from heterosexual men of color and thus provide an incomplete picture as to how the social construction and enactment of relational schemes and scripts may contribute to women’s risk for HIV infection.

## Conclusions

To minimize the potential for increasing rates of HIV infection among heterosexual women of color, namely African American and Hispanic, in the near future, greater attention needs to be focused on the scripts that influence their relationship expectations, relationship behaviors, and sexual risk taking. The availability and development of female-controlled/initiated preventative methods are imperative; however, failure to address the sociocultural norms and assumptions that frame women and men’s sexual relationships may hamper the adoption of such strategies. To ensure new biomedical interventions do not encounter challenges seen with condoms, we recommend that interventions at both the couple- and gender-specific group-level aim to take apart (deconstruct/analyze) and rewrite (reconstruct) as appropriate heterosexual relationship scripts.

## Abbreviations

AL: Alabama; FL: Florida; HIV: Human immunodeficiency virus; NC: North Carolina; STI(s): Sexually transmitted infection(s).

## Competing interests

Financial competing interests

The authors declared no potential conflicts of interest with respect to the research, authorship, and/or publication of this article.

Non-financial competing interests

The authors declare that they have no non-financial competing interests.

## Authors’ contributions

EML, CO, GM, OVL, CS, and AA were significantly involved in the design and implementation of the study; EML developed the overall analysis plan for this paper and led the interpretation of the results and writing and revising of the article; LT, OD, and GM made substantial contributions to the drafting of the manuscript, provided input on the interpretation of results, and revisions to the manuscript., EML and LT performed the qualitative data analysis; CO participated in reviewing the transcripts and developing the preliminary codebook, and in summarizing the quantitative data; OVL, CS, and AA oversaw data collection at their research sites and provided significant intellectual contribution to the finalization of the manuscript. All authors read and approved the final manuscript.

## Pre-publication history

The pre-publication history for this paper can be accessed here:

http://www.biomedcentral.com/1472-6874/13/27/prepub

## Supplementary Material

Additional file 1 Semi-structured qualitative interview guide questions, five counties in the southeastern US, 2008-09. Click here for file

Additional file 2 Thematic explanations by “if-then” relationship scenarios, qualitative interviews in five counties in the southeastern US, 2008-09. Click here for file
