# Supplementary material for: “A man’s gonna do what a man wants to do”: African American and Hispanic women’s perceptions about heterosexual relationships: a qualitative study
Source: BMC Womens Health. 2013 May 24;13:27. doi: 10.1186/1472-6874-13-27 (PMC3666901; doi:10.1186/1472-6874-13-27)
Supplement: Additional file 1 — Semi-structured qualitative interview guide questions, five counties in the southeastern US, 2008-09. [file 1472-6874-13-27-S1.docx]

Additional File 1 *Semi-structured qualitative interview guide questions, five counties in the southeastern US, 2008-2009*

Let’s start by talking about men and women in your community. How do they usually interact with one another?

*Probes:*

What types of things do they do together?

What things do they do apart?

Now, I would like to talk about relationships. What does the word relationship mean to you?

*Probes:*

In your experience, what types of relationships do men and women share?

What types of relationships are less common between them?

Let’s now focus on communication. How do men and women communicate with one another?

*Probes:*

What can a woman easily talk with a man about?

What does a woman find hard to talk to a man about?

What things do men and women see from a different point of view?

What is important to a woman in choosing a partner?

*Probes:*

Tell me what you look for in a relationship with a man?

Thinking about other women in your community, what might they look for in a relationship with a man?

In your experience, what does a man usually look for in a relationship?

What are the differences between a romantic sexual relationship and a non-romantic one?

Walk me through the steps that men and women go through to form a relationship.

Now, I want to talk about stability in relationships between men and women. What does a stable relationship look like?

*Probes:*

What helps some relationships last longer than others?

What causes some relationships to end?

Now, I would like us to talk about behavior expected of men and women. We refer to this as a role. What is a woman’s role in a relationship?

*Probes:*

How do these things come to be her responsibility?

What happens if a woman is unable to carry out her role?

What is a man’s role in a relationship?

*Probes:*

How do these things come to be his responsibility?

What happens if a man is unable to carry out his role?

What are women likely to put up with in a relationship?

*Probes:*

What are they not going to put up with?

How will a man react when he finds out that woman is not going to put up with his behavior?

I want to focus on how decisions are made in a relationship. Describe how this happens in a sexual relationship.

*Probes:*

What does the term “being in control” mean to you?

What influences how much say-so a woman has in her relationships?

Now, I want to shift our conversation to HIV. What comes to mind when you hear the words “at risk”?

*Probes:*

Whom do you see in your community being most at risk for HIV?

What do you think puts [African-American/Hispanic] women at risk for being infected with HIV?

What would help to keep them from becoming infected?
