# Supplementary material for: “A man’s gonna do what a man wants to do”: African American and Hispanic women’s perceptions about heterosexual relationships: a qualitative study
Source: BMC Womens Health. 2013 May 24;13:27. doi: 10.1186/1472-6874-13-27 (PMC3666901; doi:10.1186/1472-6874-13-27)
Supplement: Additional file 2 — Thematic explanations by “if-then” relationship scenarios, qualitative interviews in five counties in the southeastern US, 2008-09. [file 1472-6874-13-27-S2.pptx]

## Slide 1
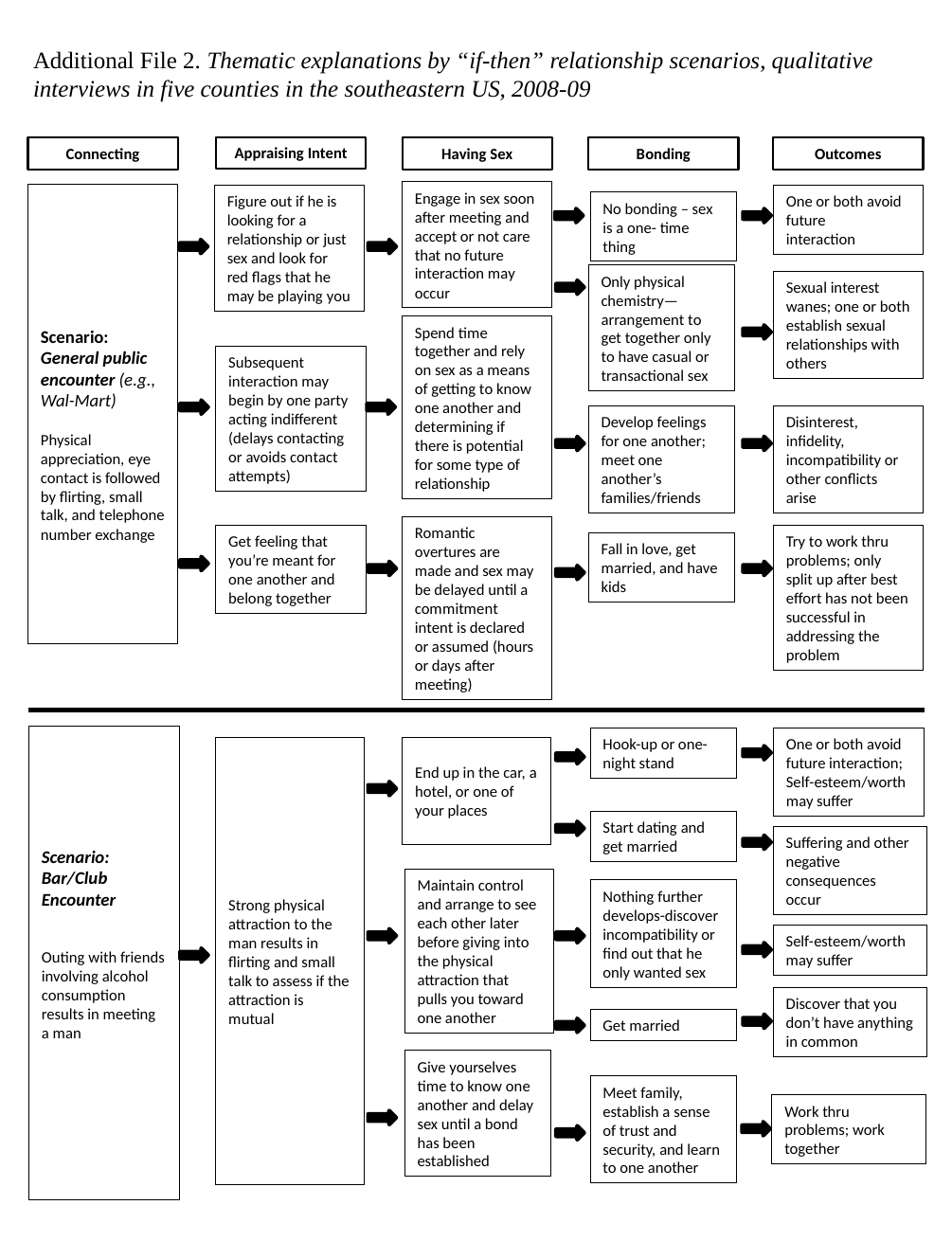

Additional File 2. Thematic explanations by “if-then” relationship scenarios, qualitative interviews in five counties in the southeastern US, 2008-09
Appraising Intent
Connecting
Having Sex
Bonding
Outcomes
Engage in sex soon after meeting and accept or not care that no future interaction may occur
Scenario: General public encounter (e.g., Wal-Mart)
Physical appreciation, eye contact is followed by flirting, small talk, and telephone number exchange
Figure out if he is looking for a relationship or just sex and look for red flags that he may be playing you
One or both avoid future
interaction
No bonding – sex is a one- time thing
Only physical chemistry—arrangement to get together only to have casual or transactional sex
Sexual interest wanes; one or both establish sexual relationships with others
Spend time together and rely on sex as a means of getting to know one another and determining if there is potential for some type of relationship
Subsequent interaction may begin by one party acting indifferent (delays contacting or avoids contact attempts)
Disinterest, infidelity, incompatibility or other conflicts arise
Develop feelings for one another; meet one another’s families/friends
Romantic overtures are made and sex may be delayed until a commitment intent is declared or assumed (hours or days after meeting)
Get feeling that you’re meant for one another and belong together
Try to work thru problems; only split up after best effort has not been successful in addressing the problem
Fall in love, get married, and have kids
Scenario: Bar/Club Encounter
Outing with friends involving alcohol consumption results in meeting a man
One or both avoid future interaction; Self-esteem/worth may suffer
Hook-up or one-night stand
Strong physical attraction to the man results in flirting and small talk to assess if the attraction is mutual
End up in the car, a hotel, or one of your places
Start dating and get married
Suffering and other negative consequences occur
Maintain control and arrange to see each other later before giving into the physical attraction that pulls you toward one another
Nothing further develops-discover incompatibility or find out that he only wanted sex
Self-esteem/worth may suffer
Discover that you don’t have anything in common
Get married
Give yourselves time to know one another and delay sex until a bond has been established
Meet family, establish a sense of trust and security, and learn to one another
Work thru problems; work together

## Slide 2
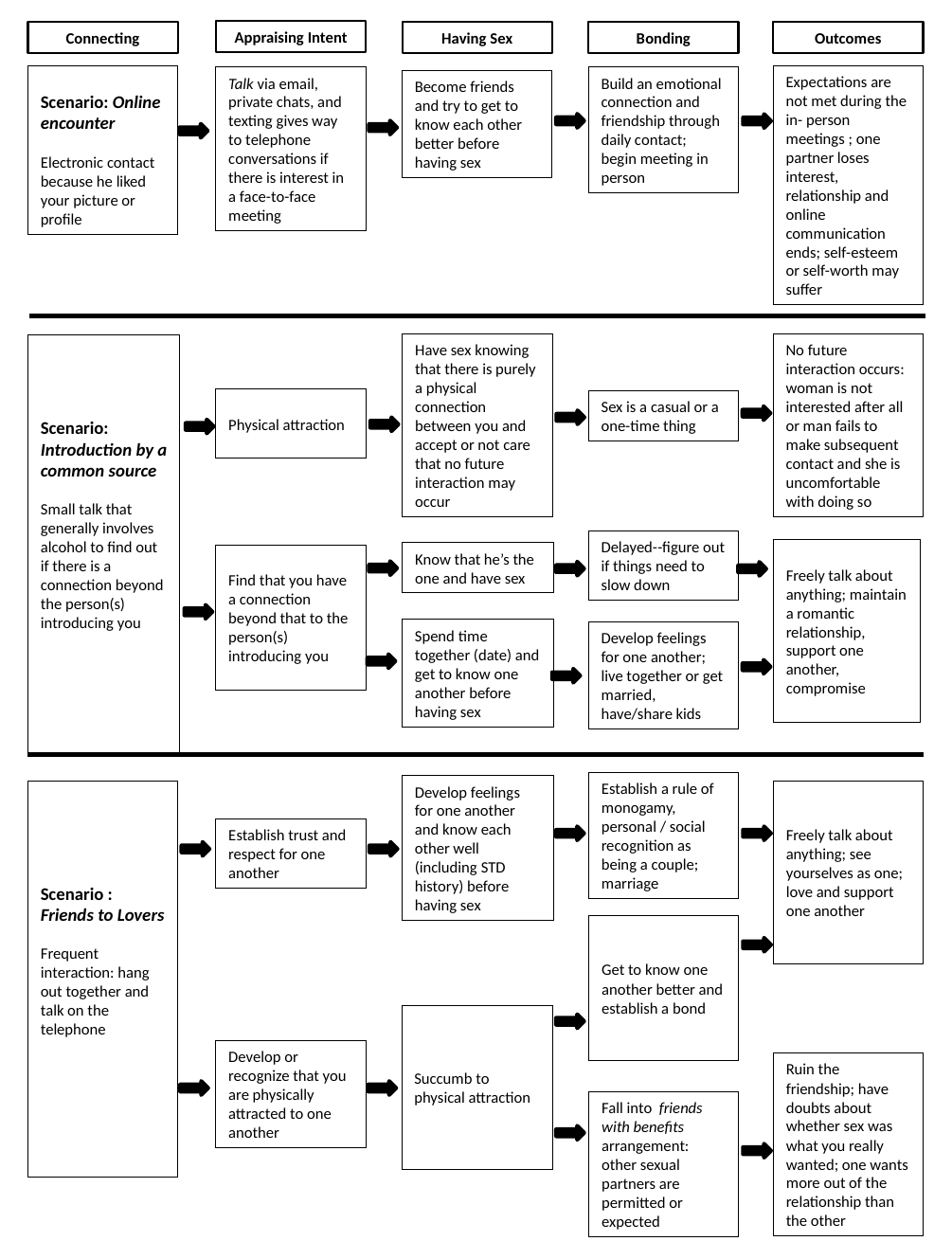

Appraising Intent
Connecting
Having Sex
Bonding
Outcomes
Scenario: Online encounter
Electronic contact because he liked your picture or profile
Expectations are not met during the in- person meetings ; one partner loses interest, relationship and online communication ends; self-esteem or self-worth may suffer
Talk via email, private chats, and texting gives way to telephone conversations if there is interest in a face-to-face meeting
Build an emotional connection and friendship through daily contact; begin meeting in person
Become friends and try to get to know each other better before having sex
Have sex knowing that there is purely a physical connection between you and accept or not care that no future interaction may occur
No future interaction occurs: woman is not interested after all or man fails to make subsequent contact and she is uncomfortable with doing so
Scenario: Introduction by a common source
Small talk that generally involves alcohol to find out if there is a connection beyond the person(s) introducing you
Physical attraction
Sex is a casual or a one-time thing
Delayed--figure out if things need to slow down
Freely talk about anything; maintain a romantic relationship, support one another, compromise
Know that he’s the one and have sex
Find that you have a connection beyond that to the person(s) introducing you
Spend time together (date) and get to know one another before having sex
Develop feelings for one another; live together or get married, have/share kids
Establish a rule of monogamy, personal / social recognition as being a couple; marriage
Develop feelings for one another and know each other well (including STD history) before having sex
Scenario : Friends to Lovers
Frequent interaction: hang out together and talk on the telephone
Freely talk about anything; see yourselves as one; love and support one another
Establish trust and respect for one another
Get to know one another better and establish a bond
Succumb to physical attraction
Develop or recognize that you are physically attracted to one another
Ruin the friendship; have doubts about whether sex was what you really wanted; one wants more out of the relationship than the other
Fall into friends with benefits arrangement: other sexual partners are permitted or expected

## Slide 3
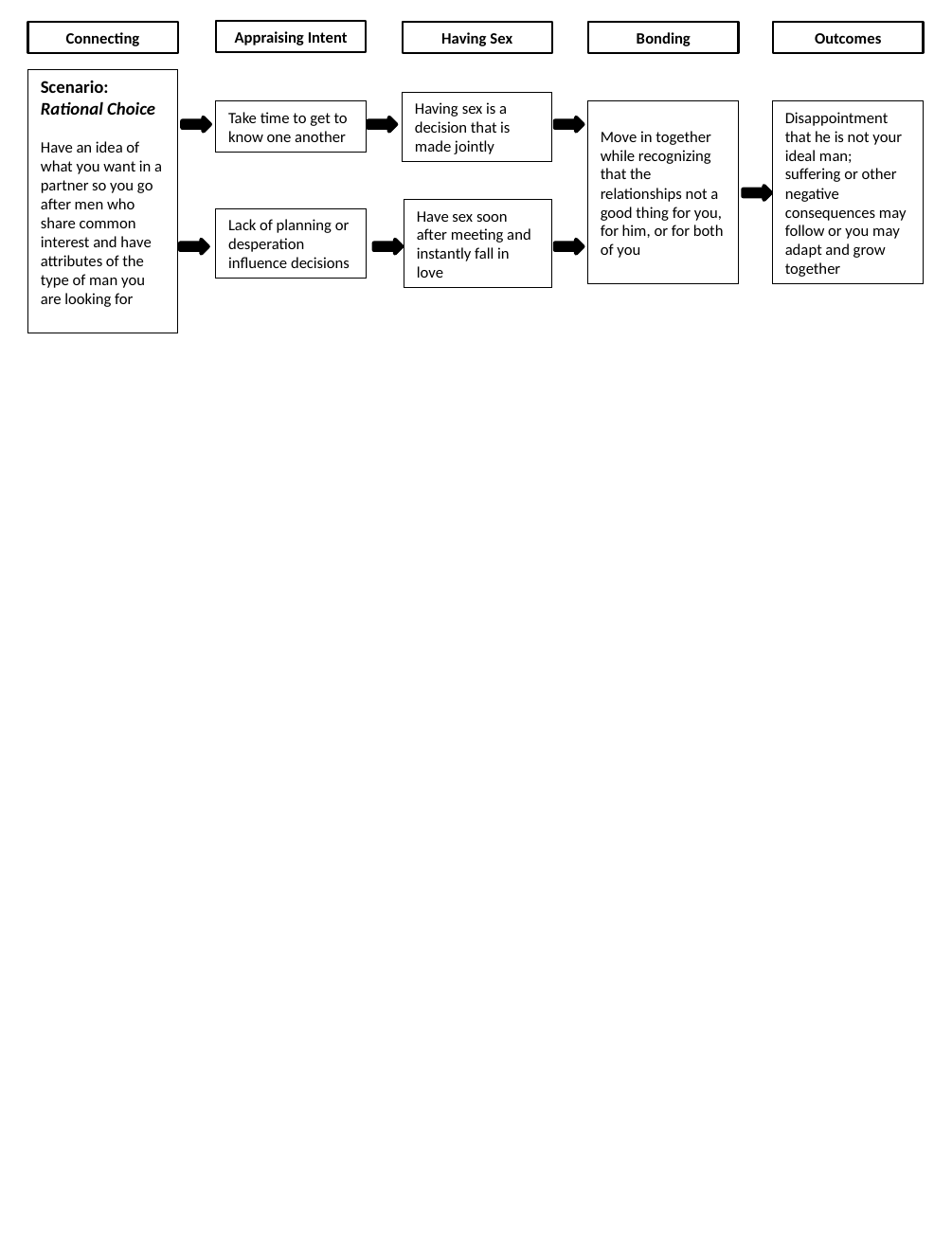

Appraising Intent
Connecting
Having Sex
Bonding
Outcomes
Scenario: Rational Choice
Have an idea of what you want in a partner so you go after men who share common interest and have attributes of the type of man you are looking for
Having sex is a decision that is made jointly
Take time to get to know one another
Move in together while recognizing that the relationships not a good thing for you, for him, or for both of you
Disappointment that he is not your ideal man; suffering or other negative consequences may follow or you may adapt and grow together
Have sex soon after meeting and instantly fall in love
Lack of planning or desperation influence decisions
